# Liver regeneration: cytokine regulation targeting hepatocytes and beyond

**DOI:** 10.1093/lifemedi/lnag004

**Published:** 2026-01-29

**Authors:** Ting Xiao, Zhangliu Jin, Jiangming Deng, Wen Meng

**Affiliations:** Department of Hepatopathy and Endocrinology, The Affiliated Children’s Hospital of Xiangya School of Medicine, Central South University (Hunan Children’s Hospital), Changsha 410007, China; Department of General Surgery, The Second Affiliated Hospital of Anhui Medical University, Hefei 230601, China; Metabolic Syndrome Research Center, The Second Xiangya Hospital of Central South University, Changsha 410011, China; Department of Oncology, The Second Xiangya Hospital of Central South University, Changsha 410011, China; Metabolic Syndrome Research Center, The Second Xiangya Hospital of Central South University, Changsha 410011, China

## Abstract

The liver, the largest glandular organ in humans, exhibits a unique and robust regenerative capacity following injury. This regenerative response is orchestrated through a highly regulated network of cellular and molecular signals. Here, we review the cytokine-mediated regulation of liver regeneration, emphasizing autocrine, paracrine, as well as endocrine pathways. Hepatocyte proliferation is modulated not only by intrinsic signals but also by cytokines derived from non-parenchymal cells—including Kupffer cells, hepatic stellate cells, and liver sinusoidal endothelial cells—as well as by endocrine cues from the systemic circulation. From the perspective of metabolic reprogramming, these regulatory pathways illustrate how adaptive changes in glucose, lipid, and amino acid metabolism collectively sustain the cellular activities essential for liver regeneration. We further explore how metabolic adaptations contribute to regeneration, providing mechanistic insights and revealing potential therapeutic targets for liver diseases. Finally, we discuss emerging strategies that target cytokine networks and metabolic pathways to enhance liver regeneration, highlighting recent advances in translational applications.

## Introduction

The incidence of liver diseases has been on a concerning upward trajectory, necessitating liver transplantation for a significant number of patients [1]. However, the scarcity of donor organs and the challenges posed by transplant rejection have become formidable barriers to effective treatment. To mitigate this supply–demand disparity, liver resection has emerged as a rapidly advancing alternative, with hepatic surgery remaining the predominant therapeutic approach for liver cancer. Post-resection, the remnant liver possesses an intrinsic capacity to regenerate, thereby restoring liver structure and function. Nonetheless, an inefficient regenerative response can compromise the liver’s ability to sustain vital functions, potentially leading to liver failure and serious complications such as small-for-size syndrome [2–4]. Consequently, a profound understanding of the mechanisms underlying liver regeneration is imperative for identifying intervention targets aimed at enhancing regenerative efficacy.

**Figure 1.**
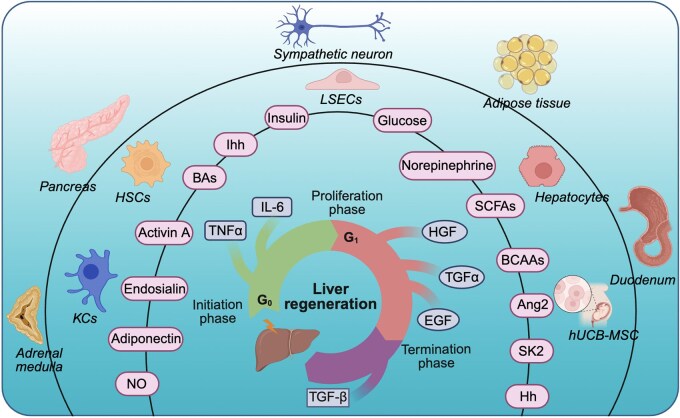
Overview of the liver regeneration process.

The regenerative process is conventionally delineated into three distinct phases: initiation, proliferation, and termination. During the initiation phase, hepatocytes transition from the G0 to the G1 phase, acquiring sensitivity to growth and transcription factors [5]. This transition is triggered primarily by the pro-inflammatory cytokines interleukin-6 (IL-6) and tumor necrosis factor alpha (TNFα). Kupffer cell (KC)-derived IL-6 activates the IL-6R/gp130 complex on hepatocytes, leading to the Janus kinase-signal transducer and activator of transcription 3 (STAT3) signaling and the induction of early-response genes, including cyclin D1. Concurrently, TNFα stimulates c-Jun N-terminal kinase (JNK)-mediated phosphorylation of c-Jun, which promotes transcription of genes such as cyclin-dependent kinase (*CDK1*) [6, 7]. These early transcriptional events are reinforced by late-response genes and growth factor signaling, establishing a robust proliferative signal. The proliferation phase is driven by growth factors including hepatocyte growth factor (HGF), epidermal growth factor (EGF) and transforming growth factor α (TGFα), which propel hepatocytes through G1 and S phase, culminating in mitosis. This phase is tightly regulated by both primary and auxiliary mitogens to ensure synchronized replication. Finally, the termination phase is initiated once liver mass is restored. Inhibitory signals, particularly TGFβ, suppress further proliferation and promote cell-cycle exit, preventing hyperplasia and re-establishing functional liver mass [6]. Liver regeneration is a complex, multi-cellular, and multi-factorial process that relies on the interplay between hepatocytes, non-parenchymal liver cells, and extra-hepatic tissues (Fig. 1). This process is modulated through a synergistic combination of autocrine, paracrine, and endocrine signaling pathways (Fig. 1). Understanding the complex interplay between these signaling pathways and how they are integrated at the cellular level to coordinate liver regeneration poses a significant challenge.

## Autocrine cytokine regulation in liver regeneration

**Figure 2.**
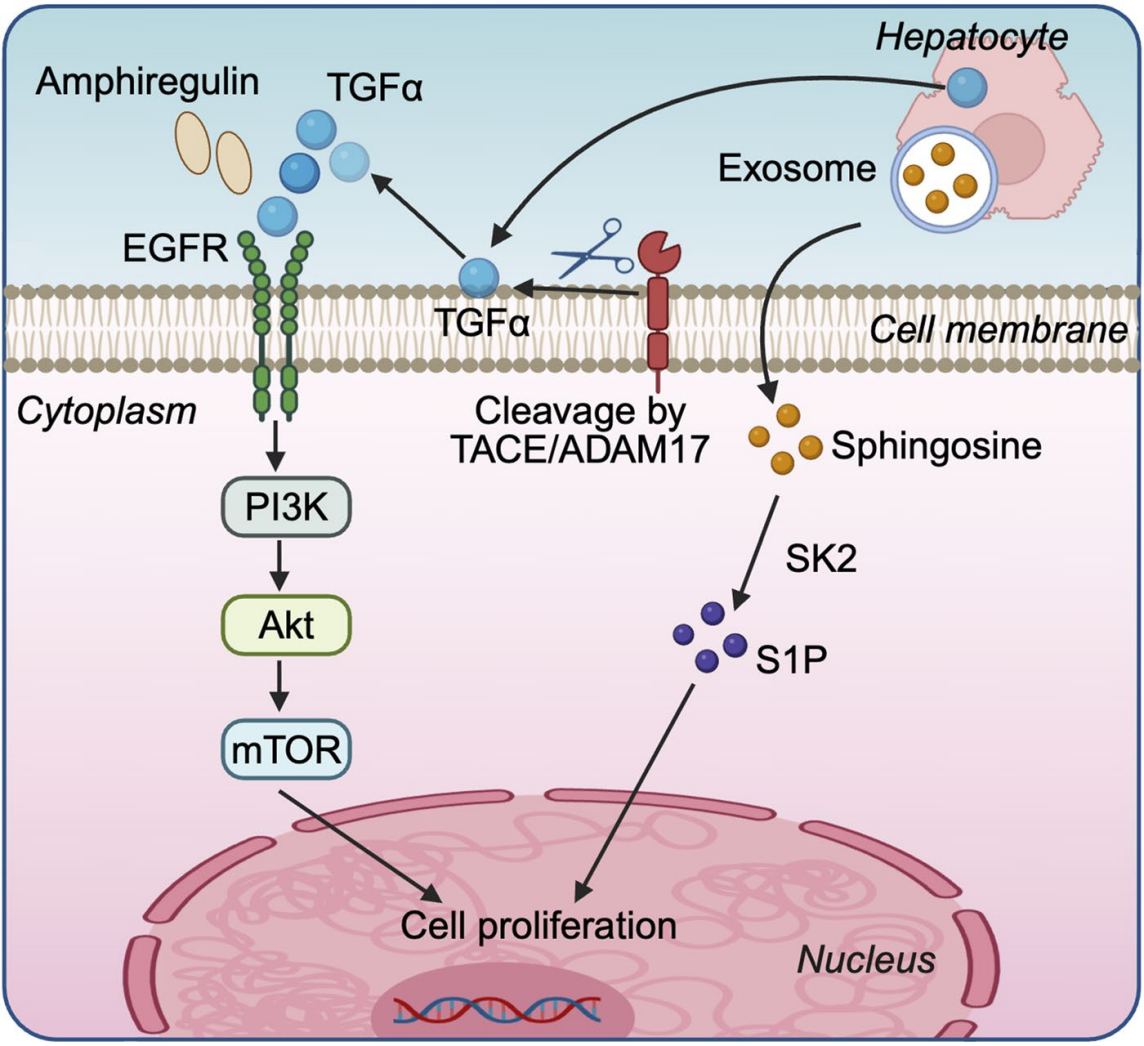
Autocrine cytokine-mediated mechanisms promoting hepatocyte proliferation during liver regeneration. Following hepatectomy, hepatocyte-derived TGFα is upregulated and cleaved by TACE/ADAM17, activating EGFR signaling to drive proliferation. Amphiregulin acts as a compensatory ligand with rapid post-injury activation. Additionally, hepatocyte-secreted exosomes enriched in SK2 promote S1P-mediated signaling to enhance regenerative responses. TGFα, transforming growth factor α; EGFR, epidermal growth factor receptor; SK2, sphingosine kinase 2; PI3K, phosphatidylinositol 3-kinase; mTOR, mammalian target of rapamycin; S1P, sphingosine-1-phosphate. The figure was created with BioRender.

In the context of liver regeneration, hepatocytes play a central role as the primary effector cells, with their proliferation being intricately regulated by a spectrum of cytokines, including those they secrete autonomously. One such cytokine is TGFα, which, while typically not present in the liver under normal conditions, is swiftly upregulated following partial hepatectomy (PH) (Fig. 2). This induction is pivotal to the subsequent regenerative process, where TGFα, initially present as a transmembrane precursor, is cleaved by the sheddase TACE/ADAM17 at the extracellular domain, thereby releasing its bioactive soluble form [8, 9] (Fig. 2). As a member of the EGF ligand family, TGFα exerts its influence by binding to the EGF receptor (EGFR) on hepatocytes, thereby initiating a cascade of intracellular signaling events conducive to cell proliferation (Fig. 2). Notably, studies have indicated that elevated levels of TGFα within the liver can stimulate hepatocyte proliferation, albeit with the potential risk of promoting tumorigenesis [10]. Conversely, the absence of TGFα in genetically modified mice has been observed not to impair the liver’s regenerative capacity [11], suggesting a compensatory mechanism involving other EGF ligands. Among these compensatory factors, amphiregulin, another member of the EGF ligand family, has been identified as a rapid inducer of liver regeneration, binding to the EGF receptor within 30 min post-hepatectomy to facilitate the regenerative process (Fig. 2). The significance of amphiregulin is underscored by the finding that its deletion in transgenic mice results in a marked suppression of liver regeneration capabilities [12]. Moreover, recent research has highlighted the role of exosomes secreted by hepatocytes in paracrine regulation of hepatocyte proliferation. These exosomes, containing sphingosine kinase 2 (SK2), can induce the production of sphingosine-1-phosphate (S1P) in target hepatocytes, thereby enhancing their proliferative capacity [13] (Fig. 2). The intricate balance between these cytokines and their receptors, as well as the compensatory mechanisms that come into play when one pathway is compromised, underscores the complexity of liver regeneration. Understanding these mechanisms is fundamental to identifying potential therapeutic targets that could augment liver regeneration, particularly in the context of liver diseases and post-surgical scenarios.

## Paracrine cytokine regulation in liver regeneration

### Effects of Kupffer cells on liver regeneration

**Figure 3.**
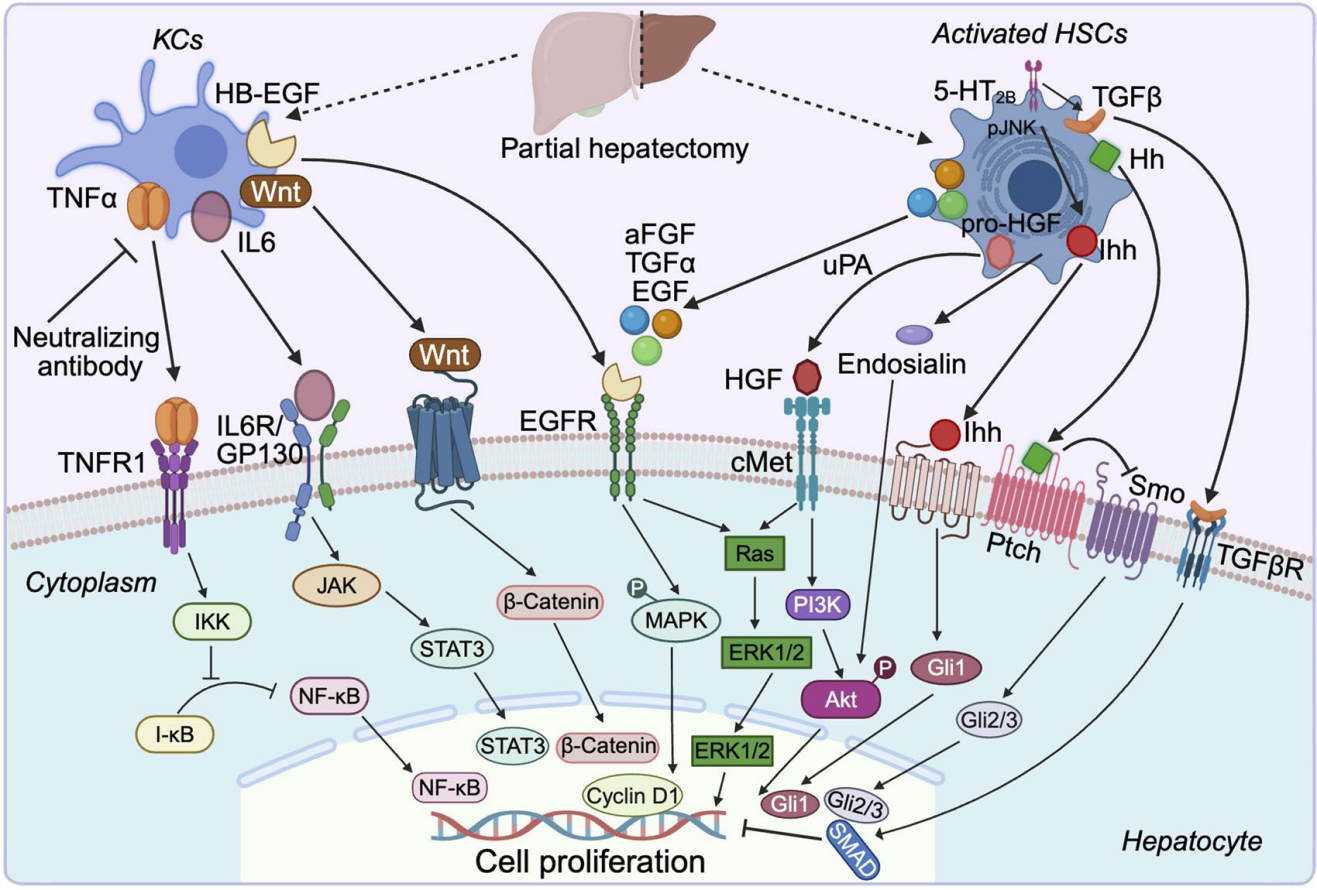
Multifaceted regulatory roles of KCs and hepatic stellate cells in liver regeneration. KCs, as liver-resident macrophages, secrete TNFα, IL-6, Wnt, and HB-EGF to promote hepatocyte proliferation through NF-κB, STAT3, β-catenin, and EGFR pathways. HSCs transition from a quiescent to an activated state after liver injury, subsequently releasing mitogenic factors such as HGF, EGF, and Hh ligands to stimulate hepatocyte proliferation via PI3K/Akt, ERK1/2, and hedgehog signaling pathways. Additionally, activated HSCs regulate the termination phase by secreting inhibitory cytokines such as TGFβ. KCs, Kupffer cells; HSCs, hepatic stellate cells; TNFα, tumor necrosis factor α; IL-6, interleukin 6; HB-EGF, heparin-binding epidermal growth factor-like growth factor; Hh, hedgehog; NF-κB, nuclear factor kappa-B; STAT3, signal transducer and activator of transcription 3; EGFR, epidermal growth factor receptor; ERK1/2, extracellular regulated protein kinases 1/2; TGFβ, transforming growth factor β; uPA, urokinase-type plasminogen activator. The figure was created with BioRender.

Kupffer cells (KCs), specialized macrophages residing in the liver that are one part of the mononuclear phagocyte system, can participate in the process of liver regeneration when the liver is injured. When the toxic agent dichloromethylenebiphosphonate (Cl_2_MDP) was used to eliminate KCs, liver regeneration was significantly impaired after PH, indicating the importance of KCs in liver regeneration [14]. As inflammatory cells in the liver, KCs secrete inflammatory factors that participate in the regulation of liver regeneration. KCs are the major source of TNFα and IL-6 in the liver. Studies have shown that neutralizing TNFα with antibodies can lead to delayed liver regeneration after PH (Fig. 3), a finding consistent with observations in TNFR1 knockout mice [15, 16]. Upon binding to its receptor TNFR1, TNFα recruits IKK kinase to the receptor complex, where it becomes activated and triggers degradation of I-kappaB (I-κB), an inhibitory partner of nuclear factor κB (NF-κB). This degradation of I-κB results in the release and translocation of NF-κB to the nucleus, where it stimulates transcription of target genes to promote cell proliferation [17–20] (Fig. 3). Plasma IL-6 levels increase after PH, and IL-6-deficiency results in impaired liver regeneration and increased liver injury [21] (Fig. 3). KCs also secrete Wnt proteins, which bind to hepatocyte receptors and activate the canonical Wnt/β-catenin signaling pathway, thereby regulating downstream genes involved in liver regeneration (Fig. 3). Yang et al. [22] found that inhibiting Wnt secretion significantly reduces cyclin D1 expression and decreases the number of hepatocytes entering the S phase after PH. KCs can also secrete heparin-binding epidermal growth factor-like growth factor (HB-EGF) in the process of liver regeneration [23] (Fig. 3). As a member of the EGFR ligand family, HB-EGF binds to the EGF receptor to exert its biological function (Fig. 3). The liver regeneration ability of transgenic mice overexpressing HB-EGF was significantly enhanced after PH [24], while HB-EGF deficiency significantly reduced liver regeneration [25]. KCs dynamically alter their cytokine expression profiles during liver regeneration, playing a regulatory role in the initiation stage and the proliferation phase.

### Effects of hepatic stellate cells on liver regeneration

Hepatic stellate cells (HSCs) are liver-specific mesenchymal cells located in the perisinusoidal space or space of Disse, connecting hepatic sinusoidal endothelial cells and hepatocytes, accounting for about 5%–8% of the resident parenchymal cells in the liver [26]. Quiescent HSCs contain lipid droplets rich in vitamin A and are the primary cells for storing retinal derivatives. Upon injury, quiescent HSCs are phenotypically activated and transdifferentiate into a myofibroblast‐like phenotype [27]. Activated HSCs express α-smooth muscle actin and collagen I [28]. The origin of HSCs is unclear because they express the marker genes of different germ layers. Studies have shown that gliotoxin-induced apoptosis of HSCs prior to PH significantly inhibits hepatocyte proliferation [29], suggesting the importance of HSCs for liver regeneration. The neurotrophin receptor p75^NTR^ is a tumor necrosis factor receptor superfamily member. HSCs cannot be activated without p75^NTR^, thereby blocking the receptor’s role in promoting hepatocyte proliferation [30], indicating that activated HSCs play a significant role in regulating the regeneration ability of the liver. Activated HSCs promote hepatocyte proliferation by secreting HGF, and serum HGF levels increase significantly in a short period after PH [31]. HGF is stored in the extracellular matrix (ECM) in a single-chain inactive form and can be activated by urokinase-type plasminogen activator to its double-chain active form [32–34] (Fig. 3). HGF plays an important role in the process of liver regeneration through binding to its receptor c-Met [35] (Fig. 3), which is a multifunctional receptor, involved in a variety of biological processes such as cell proliferation, growth, survival, and metabolism [36, 37]. HGF binding with c-Met activates extracellular signal-regulated kinase 1/2 (ERK1/2), phosphoinositide 3-kinase (PI3K) and Akt signaling, promoting hepatocyte proliferation [38, 39] (Fig. 3). In addition to HGF, acidic fibroblast growth factor, TGFα, and EGF secreted by HSCs also contribute to liver regeneration [40–42] (Fig. 3). Another HSC-derived paracrine signal is the hedgehog (Hh) ligand family (Fig. 3). Ochoa et al. [43] found that blocking the Hh pathway with cyclopamine inhibits liver regeneration, highlighting its critical role. Blocking Hh signaling in HSCs causes significant suppression of *Gli1* and *Yap1* mRNA in the neighboring hepatocytes and therefore inhibits hepatocyte proliferation [44] (Fig. 3). This study suggests that HSCs may be involved in the regulation of hepatocytes proliferation through paracrine Hh ligands (Fig. 3). Another group found that inhibition of JNK1 in HSCs could significantly reduce the secretion of Ihh, leading to a decrease in liver/body mass ratio and a significant inhibition of hepatocyte proliferation (Fig. 3). It can be seen from this that Ihh secreted by HSCs plays an important role in the regulation of liver regeneration [45]. In addition to regulating proliferation, HSCs also secrete cytokines that control the termination phase of liver regeneration, most notably TGFβ [46, 47] (Fig. 3). TGFβ activates downstream signaling pathways by binding with its receptors TGFβR1 and TGFβR2 to participate in the regulation of cell growth and apoptosis. Romero-Gallo et al. [48] used liver-specific TGFβR2 knockout mice to study its role in proliferation and found increased hepatocyte proliferation and enhanced phosphorylation of P130 after PH, suggesting that TGFβ may regulate liver regeneration through P130. Serotonin induces TGFβ secretion by activating the 5-HT_2B_ receptor on HSCs, thereby inhibiting the proliferation of hepatocytes (Fig. 3). Inhibition of 5-HT_2B_ significantly promotes liver regeneration in both acute and chronic liver injury models [49]. Bone morphogenetic protein 9, another cytokine in the TGFβ family, has been shown to be secreted by HSCs and to inhibit the proliferation of hepatocytes [50]. In addition, HSCs can secrete endosialin, a C-type lectin-like transmembrane protein, to participate in the regulation of hepatocyte proliferation (Fig. 3). Mogler et al. [51] found that endosialin knockout mice exhibit enhanced hepatocyte proliferation post-PH, indicating that HSC-derived endosialin negatively regulates liver regeneration. Collectively, HSCs contribute to both the proliferative and termination phases of liver regeneration by secreting stimulatory and inhibitory factors that modulate hepatocyte proliferation.

### Effects of liver sinusoidal endothelial cells on liver regeneration

**Figure 4.**
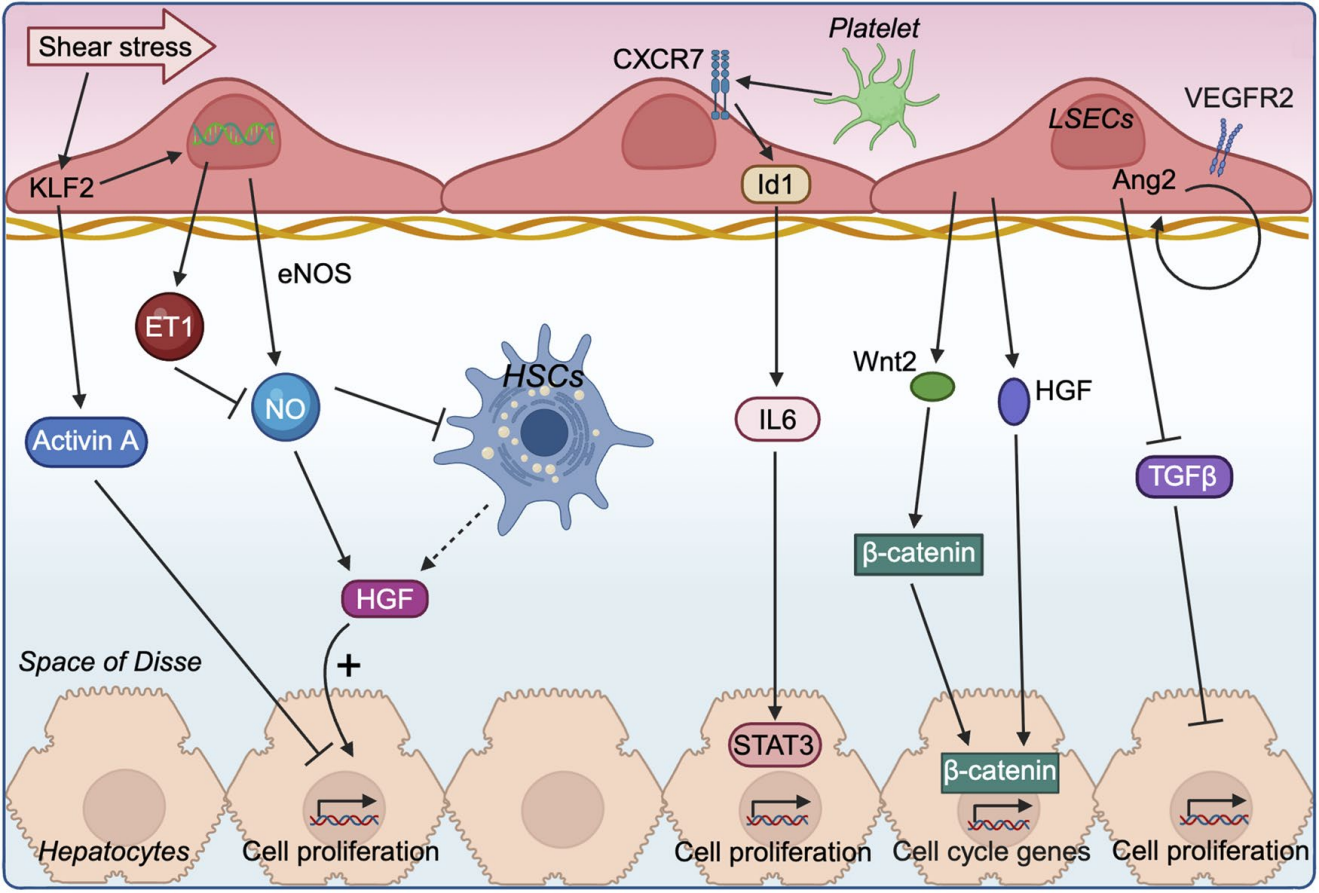
Dual regulatory roles of LSECs during liver regeneration.In response to increased shear stress after hepatectomy, LSECs secrete NO, HGF, and Wnt2 to promote hepatocyte proliferation via enhanced growth factor sensitivity and β-catenin activation. Concurrently, LSECs regulate regeneration resolution by secreting inhibitory factors such as activin A and dynamically modulating Ang2 to control TGFβ expression and endothelial remodeling. Co-culture of platelets with LSECs induces IL-6 secretion, which promotes hepatocyte proliferation. LSECs, liver sinusoidal endothelial cells; NO, nitric oxide; HGF, hepatocyte growth factor; Ang2, angiopoietin 2; TGFβ, transforming growth factor β; IL-6, interleukin 6. The figure was created with BioRender.

Liver sinusoidal endothelial cells (LSECs) are highly specialized endothelial cells. They form the wall of liver sinusoids and account for about 15%–20% of liver cells [52–54]. They possess a discontinuous structure called “fenestrae.” LSECs are located between blood cells and hepatocytes, as well as HSCs. On one side, they are exposed to mixed arterial and portal blood rich in oxygen and nutrients, while on the other side, they interact with hepatocytes and HSCs, both of which are crucial for metabolic processes [55]. The blood flow to the remaining liver increases immediately after PH, which increases the shear stress on LSECs. Under this condition, LSECs can release nitric oxide (NO) to increase the sensitivity of hepatocytes to HGF, which promotes hepatocyte proliferation [54, 56–58] (Fig. 4). LSECs can also secrete growth factors to promote hepatocyte proliferation, such as HGF and Wnt2 [59] (Fig. 4). Wnt2 regulates liver regeneration by promoting the nuclear translocation of β-catenin, thereby upregulating the expression of cell cycle-related genes and enhancing hepatocyte proliferation [60–63] (Fig. 4). The expression of angiopoietin-2 (Ang2) in LSECs is dynamically regulated after PH. During the early phase, Ang2 expression rapidly decreases, which reduces TGFβ secretion, a known inhibitor of liver regeneration, thereby promoting hepatocyte proliferation via paracrine signaling (Fig. 4). In the later phase, increased Ang2 negatively controls the liver regeneration by regulating LSEC proliferation through upregulation of vascular endothelial growth factor receptor 2 [64]. Activin A, a member of the TGFβ family, is induced by Krϋppel-like factor 2, a shear-stress-responsive transcription factor (Fig. 4). The significantly increased expression and secretion of activin A from LSECs can inhibit hepatocyte proliferation [65] (Fig. 4). Co-culture of platelets with LSECs induces IL-6 secretion from LSECs, and the secreted IL-6 promotes hepatocyte proliferation [66] (Fig. 4).

## Endocrine cytokine regulation in liver regeneration

**Figure 5.**
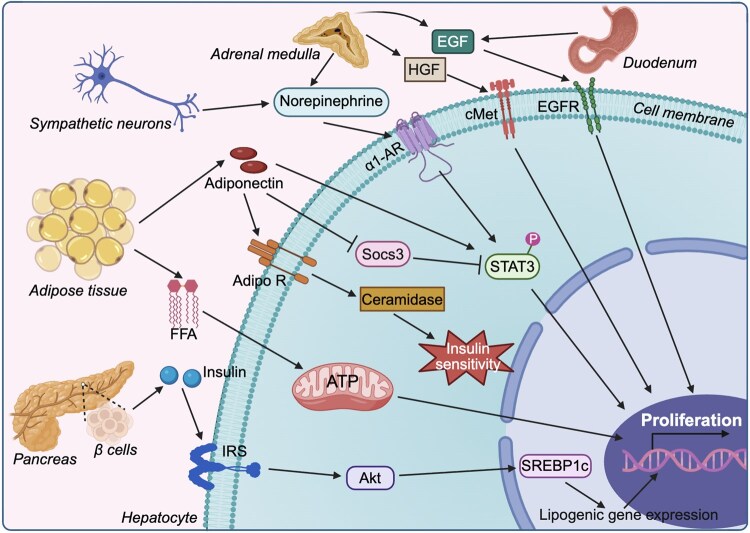
Endocrine regulation of liver regeneration via metabolic and cytokine-mediated signaling. Norepinephrine released from sympathetic neurons and the adrenal medulla promotes hepatocyte proliferation indirectly by activating α1-AR and enhancing HGF and EGF synthesis. Insulin enters the liver through the portal vein and supports regeneration by enhancing hepatocyte lipid accumulation via the Akt-SREBP1c pathway, thereby providing metabolic energy for proliferation. Adiponectin secreted by adipose tissue improves hepatic insulin sensitivity and promotes STAT3 activation, facilitating regeneration. Transient hepatic steatosis during early liver regeneration results from adipose-derived fat uptake, providing ATP through fatty acid oxidation to fuel regenerative processes. Duodenum-derived EGF reaches the liver via portal circulation and stimulates hepatocyte proliferation. α1-AR, α1-adrenergic receptors; HGF, hepatocyte growth factor; EGF, epidermal growth factor; STAT3, signal transducer and activator of transcription 3; IRS, insulin receptor substrates. The figure was created with BioRender.

Liver regeneration is regulated not only by autocrine signaling from hepatocytes and paracrine signaling from non-parenchymal cells but also by endocrine mechanisms. Norepinephrine, produced by terminal synapses of sympathetic neurons, acts as a neurotransmitter in the sympathetic nervous system and is also secreted into the blood by the adrenal medulla (Fig. 5). The concentration of norepinephrine increases after PH, and its dynamics are similar to those of HGF. Clinically used adrenergic receptor inhibitors can delay the process of liver regeneration [31, 67–69]. Although norepinephrine itself has no mitogenic activity, it can promote hepatocyte proliferation by binding to the alpha 1-adrenergic receptor (α1-AR), thereby activating STAT3 [70] (Fig. 5). It can also promote the synthesis and secretion of growth factors HGF and EGF, thereby contributing to the regulation of liver regeneration [68, 69] (Fig. 5). Insulin, a hormone synthesized by pancreatic beta cells, plays a crucial role in regulating hepatic metabolism. It enters the liver through the portal vein circulation. Studies have shown that after portacaval shunt, insulin could not enter the liver through the portal vein, resulting in liver atrophy. Upon re-injection of insulin, the liver can regain its mass. Although the precise mechanism remains unclear, it has been confirmed that insulin is involved in the regulation of liver regeneration [71]. Transient hepatic steatosis occurs in the liver at the early stage of liver regeneration, wherein fat is released from adipose tissue and increased fatty acid uptake leads to fat deposition in the liver [72–74]. Fatty acids are oxidized to produce adenosine triphosphate (ATP), which supplies energy for liver regeneration (Fig. 5). Insulin can increase lipid synthesis in hepatocytes, possibly through the serine–threonine kinase Akt/protein kinase B to induce sterol regulatory element-binding protein 1c (SREBP1c), which is responsible for inducing lipogenic gene expression and promoting lipid accumulation [75–78] (Fig. 5). Studies have shown that Akt-deficient mice exhibit reduced lipid droplets in hepatocytes, resulting in impaired proliferation [79, 80]. These findings suggest that insulin may promote liver regeneration by increasing lipid accumulation. Adiponectin is an adipokine specifically secreted by adipocytes into the circulation. It binds with its receptors to improve the insulin sensitivity in the liver. Studies have demonstrated that adiponectin deficiency impairs liver regeneration by reducing STAT3 phosphorylation and increasing suppressor of cytokine signaling 3 (SOCS3) transcription [81] (Fig. 5). EGF is mainly produced by Brunner glands of the duodenum, which enters the liver through the portal circulation to promote liver regeneration [69, 82] (Fig. 5).

## Metabolism and liver regeneration

**Figure 6.**
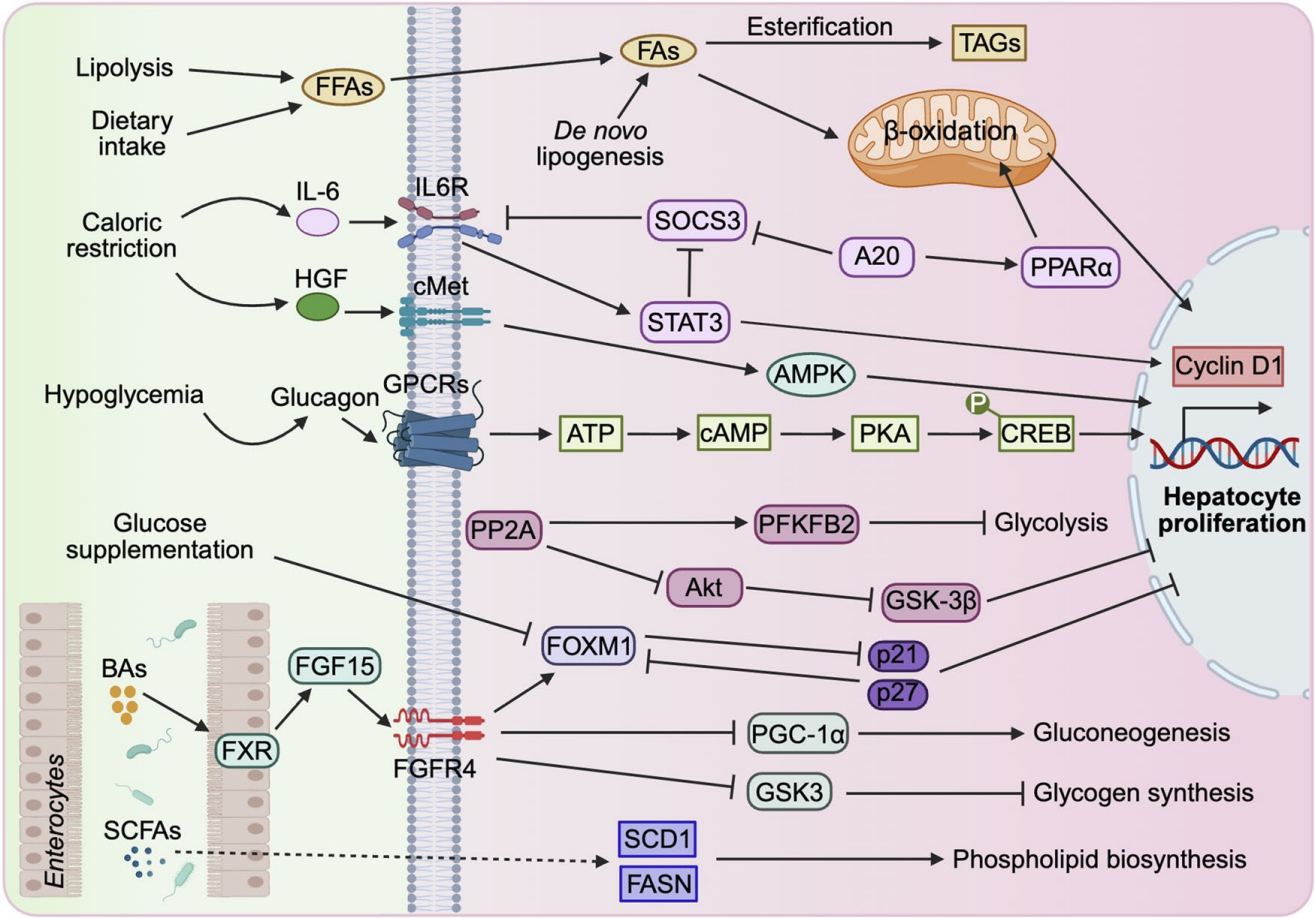
Metabolic reprogramming orchestrates liver regeneration. During liver regeneration, nutritional status serves as a critical regulator: caloric restriction promotes the expression of pro-mitogenic mediators including IL-6 and HGF, whereas glucose supplementation suppresses cell cycle progression via induction of p21/p27 and downregulation of FOXM1. BAs signaling through the FXR receptor orchestrates regenerative processes along the gut–liver axis, activating hepatic FOXM to drive cell cycle progression while stimulating the intestinal FGF15/FGFR4 pathway, which concurrently fine-tunes hepatic glucose homeostasis. Meanwhile, free fatty acids from various metabolic sources support regeneration through β-oxidation for energy production or esterification into triglycerides for membrane biosynthesis. Gut microbiota-derived SCFAs are directly incorporated into phospholipids and enhance hepatocyte proliferation by upregulating key lipogenic enzymes including SCD1 and FASN. Furthermore, the zinc finger protein A20 facilitates STAT3-mediated proliferative signaling through suppression of SOCS3. The termination phase of regeneration is ultimately mediated by PP2A, which attenuates proliferative drive by suppressing the Akt/GSK-3β/cyclin D1 axis and inhibiting glycolytic flux via PFKFB2, thereby ensuring the return to metabolic homeostasis upon completion of the regenerative process. BAs, bile acids; FXR, farnesoid X receptor; CYP7α1, 7α-hydroxylase; FOXM1, forkhead box M1; FFA, free fatty acids. SCD1, stearoyl-CoA desaturase 1; FASN, fatty acid synthase. The figure was created with BioRender.

The liver serves as a central regulator of multiple metabolic pathways, including glucose, lipid, bile acids (BAs), and amino acid metabolism. Concurrently, metabolites and metabolic intermediates themselves function as signaling molecules that modulate key pathways in liver regeneration [83]. In the early stages of liver regeneration, lipids accumulate in the liver to provide energy for liver regeneration. Alterations in lipid metabolism play a crucial role in liver regeneration following PH [84]. Fatty acids (FAs) utilized during this process originate from multiple sources: *de novo* lipogenesis, autophagy-mediated release from lysosomal reservoirs, and active uptake from the circulating free FAs pool (Fig. 6). Depending on metabolic demands, these FAs are either esterified into triacylglycerols for storage or rapidly channeled into oxidative pathways (Fig. 6). Notably, β-oxidation serves as the primary energy source during fasting states and is indispensable for sustaining regenerative homeostasis [85]. Impaired β-oxidation is associated with suppressed regenerative capacity, whereas abundant lipid stores can adequately fuel the regeneration process through efficient oxidation, highlighting the essential role of lipid metabolism in liver repair [86]. Accumulated lipids not only provide energy and substrates for membrane biosynthesis but also function as signaling molecules that modulate downstream metabolic pathways, thereby coordinating hepatocyte proliferation through transcriptional mechanisms [84]. Notably, lipid remodeling contributes to a permissive metabolic and inflammatory microenvironment that facilitates compensatory proliferation in metabolic dysfunction-associated steatohepatitis [87]. Insulin promotes the storage of macromolecules by stimulating lipogenesis, protein synthesis, and glycogenesis, while inhibiting glycogenolysis and lipolysis [88]. Akt acts as a major downstream effector of insulin signaling and induces hepatic SREBP1c [79]. Akt-deficient mice exhibit impaired glycogenesis, reduced cell proliferation and hypertrophy, and diminished lipid droplet formation, all of which compromise liver regeneration [80]. S1P, a bioactive sphingolipid metabolite, plays a critical role in the precise regulation of sinusoidal vessel assembly during regeneration. Activation of the endothelial S1P receptor by its natural ligand or a synthetic agonist promotes liver regeneration. S1P exerts antiapoptotic effects on LSECs and stimulates hepatocyte proliferation in a paracrine manner [89]. EGF and HGF regulate the termination of the regeneration-associated steatosis (TRAS) by activating their respective receptors, EGFR and c-Met, which in turn modulate key transcription factors involved in lipolysis and fatty acid biosynthesis [90]. Intriguingly, impaired lipid metabolism resulting from EGFR loss can be compensated by MET signaling, thereby preserving normal regenerative outcomes [90]. Further studies revealed that both HGF and EGF can induce nuclear factor erythroid 2-related factor 2 (NRF2) expression, leading to the transcriptional activation of the parkinsonism-associated deglycase (*PARK7*) gene [91]. Deficiency in PARK7 impairs fatty acid β-oxidation and reduces ATP production via suppression of peroxisome proliferator-activated receptorα (PPARα) and carnitine palmitoyltransferase 1α in a PTEN-dependent manner [92]. These metabolic defects prolong TRAS and delay liver regeneration after PH [91–93]. The zinc finger protein A20 significantly influences lipid metabolism by inhibiting lipogenesis and promoting lipolysis [94]. It upregulates *PPARα*, a gene essential for liver regeneration, as demonstrated by the impaired regenerative capacity in PPARα-deficient mice [95] (Fig. 6). Furthermore, A20 enhances proliferative responses after hepatectomy by downregulating SOCS3, a negative regulator of IL-6/STAT3 signaling [94] (Fig. 6).

The liver plays a central role in maintaining systemic glucose homeostasis, and its capacity to regulate glucose metabolism is critically implicated in the process of liver regeneration [87]. Following PH, hypoglycemia emerges as an early metabolic adaptation that actively facilitates liver repair [87, 96]. Post-PH hypoglycemia enhances the expression of cyclin D1 and triggers the production of glucagon and catecholamines, which activate adenylate cyclase via G-protein-coupled receptors [97]. Subsequent protein kinase A activation leads to phosphorylation of cAMP response element-binding protein, promoting acetylation of the cyclin D1 promoter and supporting regenerative proliferation (Fig. 6). Artificially delaying post-PH hypoglycemia impairs this reprogramming and disrupts regeneration [97]. Liver regeneration is delayed when dextrose is used to postpone PH-induced hypoglycemia [97]. Mechanistically, exogenous glucose supplementation suppresses liver regeneration by elevating the expression of cell-cycle inhibitors p21 and p27, while reducing forkhead box protein M1 (FOXM1), a transcription factor that normally represses p21. This reciprocal repression between p21 and FOXM1 forms a regulatory circuit that finely tunes hepatocyte proliferation [97–100] (Fig. 6). Dietary caloric restriction induces the expression of pro-mitogenic mediators such as IL-6 and HGF, thereby driving hepatocyte proliferation and liver regeneration (Fig. 6). Conversely, caloric restriction improves insulin sensitivity, which further supports the regenerative process [101]. Akt, a serine/threonine kinase involved in cell size regulation, also contributes critically to PH-induced hypertrophy via regulation of the mechanistic target of rapamycin complex 1 (mTORC1) [102]. Alanine serves as a key substrate for gluconeogenesis during regeneration. Annexin A6, a calcium-dependent phospholipid-binding protein, promotes alanine uptake. Its deficiency compromises gluconeogenesis by suppressing phosphoenolpyruvate carboxykinase, leading to energy shortage and impaired regeneration [103]. Glycogen synthesis also contributes to liver repair. Cyclin-dependent kinase 5 regulatory subunit-associated protein 3 promotes glycogen synthesis, and its liver-specific knockout delays regeneration [104]. Additionally, protein phosphatase 2A (PP2A), a serine/threonine phosphatase, is upregulated during the termination phase of regeneration and modulates the Akt/GSK-3β/cyclin D1 pathway [105] (Fig. 6). PP2A further inhibits glycolysis via 6-phosphofructo-2-kinase/fructose-2,6-bisphosphatase-2 (PFKFB2), helping to cease regenerative activation [106] (Fig. 6). These findings highlight a sophisticated crosstalk between metabolic reprogramming and cell-cycle control, ensuring that energy and biosynthetic resources are efficiently allocated to drive successful liver regeneration.

During liver regeneration, amino acids serve as essential substrates for protein synthesis and metabolic precursors that fuel hepatocyte proliferation. Glutathione (GSH), a critical tripeptide composed of glutamate, cysteine, and glycine, is markedly upregulated prior to DNA synthesis and supports regeneration through its roles in redox homeostasis, detoxification, and thiol-dependent signaling [107]. Depletion of GSH delays cell cycle progression, underscoring its necessity in regenerative proliferation [108]. Glutamine, generated by glutamine synthetase, contributes to GSH synthesis and enters the tricarboxylic acid cycle via conversion to α-ketoglutarate, thereby supporting energy production and biosynthetic pathways [109, 110]. The cystine/glutamate antiporter system Xc^−^, encoded notably by Slc7a11, is strongly induced during regeneration and supplies cysteine, a rate-limiting precursor for GSH synthesis [111]. Recent TRAP-seq profiling identified this transporter as a key driver of regenerative growth, and dietary glutamine supplementation has been shown to enhance protein accumulation and accelerate liver repair [112]. Branched-chain amino acids (BCAAs), particularly valine, promote protein synthesis and facilitate hepatic recovery [113]. In contrast, aromatic amino acids appear non-essential in this process [113]. Additionally, tryptophan supports regeneration through its conversion to serotonin by tryptophan hydroxylase. Serotonin, in turn, facilitates the initiation of liver regeneration and modulates lipid metabolism via enterohepatic signaling [99]. Overall, specific amino acid metabolic pathways including glutathione synthesis, glutamine anaplerosis, BCAA utilization, and serotonin production, cooperatively orchestrate a metabolic environment conducive to successful liver regeneration.

Bile acids are specific metabolites produced by hepatocytes, and their levels are precisely regulated. Increased BAs levels activate the farnesoid X receptor (FXR), which down-regulates cholesterol 7α-hydroxylase (CYP7α1), establishing a negative feedback loop for BAs synthesis. BAs increase after PH, and reducing BAs levels by using the BA sequestrant cholestyramine (resin) impairs liver regeneration [114]. In clinical practice, liver regeneration efficiency is positively correlated with serum BA levels. Patients undergoing external biliary drainage after hepatectomy exhibit impaired liver regeneration [115]. BAs usually exert their biological effects by binding with FXR. FXR signaling orchestrates liver regeneration by the gut–liver axis. FXR can directly activate forkhead box M1b, a key regulator of cell cycle progression, thereby promoting liver regeneration [116, 117]. Intestinal FXR activation triggers FGF15/19 production, which then acts on hepatocytes by activating FGFR4 to stimulate regeneration [118] (Fig. 6). The same pathway concurrently regulates energy homeostasis, inhibiting hepatic glycogen synthesis through GSK-3 and promoting gluconeogenesis via PGC-1α [119, 120] (Fig. 6). FXR also regulates BA levels through negative feedback to prevent BAs overload from causing liver damage, thereby promoting liver regeneration [121].

Short-chain fatty acids (SCFAs), derived from gut microbial fermentation, play multifaceted roles in various physiological processes [122]. In the liver, SCFAs, particularly acetate, propionate, and butyrate, are transported via the portal vein and serve as critical substrates for *de novo* lipogenesis, supporting both hepatocyte proliferation and systemic lipid metabolism [123, 124]. Acetate, the most abundant SCFA, acts as a fundamental building block for saturated fatty acid synthesis catalyzed by fatty acid synthase (FASN). Moreover, bacterial SCFAs significantly contribute to phospholipid biosynthesis, which is essential for membrane formation during cell replication (Fig. 6). Isotope labeling studies have confirmed that microbiota-derived SCFAs are directly incorporated into phospholipids and enhance hepatocyte proliferation following PH [125]. Mechanistically, SCFAs upregulate key enzymes involved in fatty acid biosynthesis, such as stearoyl-CoA desaturase 1 and FASN [125] (Fig. 6). Beyond anabolic support, SCFAs also modulate immunometabolic homeostasis during liver regeneration [126]. By enhancing intestinal barrier function, SCFAs reduce the translocation of lipopolysaccharide and other pro-inflammatory mediators to the liver, thereby attenuating sterile inflammation [126, 127]. For instance, acetate modulates inflammatory responses in macrophages in a dose-dependent manner, likely through alterations in intracellular lipid metabolism [128]. The interplay between SCFA-driven metabolic remodeling and immune regulation offers promising therapeutic avenues for promoting liver regeneration.

The global prevalence of obesity and metabolic syndrome has risen dramatically, contributing to significant alterations in hepatic lipid and glucose metabolism [129]. These metabolic disturbances are closely linked to the pathophysiology of metabolic dysfunction-associated steatotic liver disease and impaired liver regeneration [130, 131]. Excessive lipid accumulation in hepatocytes promotes reactive oxygen species (ROS) overproduction, endoplasmic reticulum stress, and lipotoxicity, all of which impair liver regeneration and repair. Moreover, a key factor in steatotic progression is mitochondrial dysfunction, which arises from lipid overload and substantially diminishes the liver’s regenerative capacity [132, 133]. Excessive fatty acid influx into hepatocytes leads to mitochondrial overload, resulting in incomplete β-oxidation and accumulation of ROS [134, 135]. Persistent oxidative stress compromises mitochondrial integrity and function, a key initiating event in the cascade of irreversible hepatocyte damage. Consequently, impaired ATP production and overall energy failure in steatotic hepatocytes represent major factors underlying the compromised regenerative response in fatty livers [136–138]. Eventually, intrahepatic lipid deposition and obesity reduce hepatic blood flow through direct mechanical compression and systemic hypercatecholaminemia. These changes further suppress mitochondrial function, enhance ROS formation, activate KCs, and promote hepatic inflammation and apoptosis, collectively exacerbating regenerative failure [139]. Beyond obesity, diabetes, a chronic metabolic disorder with rapidly increasing incidence, also severely affects liver regeneration. Insulin resistance, the primary driver of hyperglycemia and compensatory hyperinsulinemia, underlies the blunted regenerative response [140, 141]. Studies in streptozotocin-induced diabetic rats undergoing PH have shown reduced levels of proliferating cell nuclear antigen and cyclin D1, indicating impaired cell cycle entry [142]. Diabetes exacerbates oxidative stress by increasing free radical generation and diminishing antioxidant defenses, leading to lipid peroxidation and DNA damage. In diabetic models, elevated hepatic ROS production under hyperglycemic conditions is further aggravated after hepatectomy. This oxidative stress disrupts protein, carbohydrate, and lipid metabolism, amplifies inflammatory cascades, and ultimately disrupts the regenerative process [143].

Macrophages, as key constituents of the mononuclear phagocyte system, play essential roles in innate and adaptive immunity through phagocytosis and antigen presentation [144]. Hepatic macrophages were previously considered synonymous with KCs. It is now understood, however, that liver macrophages comprise functionally heterogeneous populations with distinct developmental origins [145]. KCs originate from yolk sac erythromyeloid progenitors and are self-renewing tissue-resident macrophages, whereas monocyte-derived macrophages (MoMFs) arise from circulating monocytes that infiltrate the liver during injury [145]. A defining feature of macrophages is their plasticity and heterogeneity, enabling them to undergo phenotypic switching in response to local environmental cues. Irrespective of origin, hepatic macrophages have been broadly categorized into pro-inflammatory (M1) and anti-inflammatory (M2) polarization states, dictated by specific stimuli [146]. Classical activation by IFN-γ and toll-like receptor ligands polarizes macrophages toward an M1 phenotype via NF-κB and STAT signaling, driving pro-inflammatory cytokine production and major histocompatibility complex class II expression [147]. In contrast, alternative activation by cytokines such as IL-4, IL-10, and IL-25 promotes the M2 phenotype, which is associated with anti-inflammatory cytokine release and phagocytic clearance of cellular debris [147]. M2 macrophages are further subdivided into M2a, M2b, M2c, and M2d subsets, each characterized by distinct surface markers, secretory profiles, and immune functions. In liver regeneration, M1-derived cytokines predominantly mediate early-phase repair and antimicrobial responses, whereas M2-derived cytokines such as IL-4, IL-10, and IL-13 facilitate late-stage tissue repair, inflammation resolution, and immune tolerance [148].

The balance between M1 and M2 macrophage polarization critically influences the regenerative outcome in the liver. An increased abundance of M2 macrophages correlates with enhanced regeneration after PH [149]. Infusion of M1-polarized bone marrow-derived macrophage (M1-BMDM) exacerbated M1 polarization and ceramide accumulation, aggravating hepatocyte apoptosis and liver dysfunction post-hepatectomy. Conversely, M2-BMDM infusion promoted M2 polarization and S1P production, alleviating liver injury and stimulating hepatocyte proliferation [150]. Myeloid-specific deletion of p38α similarly favored M2 polarization, suppressed pro-inflammatory cytokine secretion, and ameliorated acute liver injury while promoting hepatocyte proliferation [151]. PPARα deficiency in myeloid cells enhanced IL-6 expression via M1 polarization, leading to STAT3 activation in hepatocytes and accelerating liver regeneration after PH [152]. Emerging evidence implicates chemokines in macrophage polarization during liver repair. Genetic or pharmacological inhibition of C–C motif chemokine ligand 5 (CCL5) promotes liver repair by skewing macrophages toward an M2 phenotype while suppressing M1 polarization [153, 154].

Activated HSCs serve as a major source of cytokines that orchestrate liver regeneration, participating in both its initiation and termination [155, 156]. Beyond cytokine secretion, activated HSCs promote regeneration by producing angiogenic factors, modulating the proliferation of endothelial cells and hepatocytes, and remodeling the ECM, which is essential for maintaining the 3D architecture of regenerating tissue [157–159]. Recent studies have identified epimorphin as another key mediator produced by HSCs during both quiescence and late-stage regeneration, where it supports morphogenesis and architectural restoration [160, 161]. Metabolic reprogramming through HSC interactions with other cell types further contributes to the regenerative process. In addition, HSCs contribute to inflammatory and vascular regulation. They modulate KC infiltration and activation via CCL2 signaling and are recruited to blood vessels through a process largely driven by platelet-derived growth factor, where they promote vascular remodeling in coordination with LSECs [162–165]. Structurally, each HSC wraps around multiple sinusoids and regulates sinusoidal blood flow during regeneration, further highlighting their hemodynamic influence [166]. Notably, HSCs exhibit cellular plasticity under specific conditions, with the potential to transdifferentiate into hepatocytes and bile duct cells, thereby directly replenishing liver parenchyma [167]. This process is mediated by the Hippo/Yes-associated protein signaling pathway [168]. Recent evidence also reveals a crucial role for HSC senescence in regeneration. This process, which is primarily triggered by CCN1, a molecule significantly upregulated following PH, operates through IL-6 and CXCL2 signaling pathways to facilitate the regenerative response [169].

## Advancing therapeutic strategies for liver disease: from molecular insights to clinical translation

The development of effective therapies for liver diseases, including acute injury, chronic damage, and liver failure, faces considerable challenges, particularly in bridging the gap between animal models and human clinical outcomes. To improve translational relevance, future research should prioritize rigorous preclinical models that better recapitulate human pathophysiology, such as humanized animal systems, patient-derived organoids, and bioengineered liver platforms [170]. These tools will help elucidate the mechanisms of liver regeneration and accelerate the development of clinically viable interventions.

Metabolic reprogramming has emerged as a key regulator of liver repair, where specific nutrients and metabolites influence not only energy supply but also signaling pathways governing cell proliferation and inflammation [171, 172]. Targeting cell-type-specific metabolic pathways—informed by dynamic profiling of metabolic changes during regeneration—represents a promising therapeutic avenue [173–175].

Gene therapy offers another powerful approach, with CRISPR-Cas9 enabling precise genetic modification and epigenetic editing [176–179]. While its versatility is well established, concerns regarding off-target effects and delivery efficiency remain. Future efforts should focus on optimizing tissue-specific delivery systems and enhancing spatiotemporal control of gene editing activity *in vivo* [180, 181].

Cell-based therapies, particularly those using mesenchymal stem cells (MSCs), have shown promise in treating fibrosis and liver failure. MSCs exhibit low immunogenicity, high plasticity, and regenerative paracrine activity. After transplantation, they home to injured sites, promote hepatocyte proliferation, enhance fatty acid oxidation via mTOR activation, and secrete protective factors such as IL-10 [182–186]. Extracellular vesicles and exosomes derived from MSCs also exhibit regenerative and anti-fibrotic effects [187, 188]. Nevertheless, in cell therapy trials, key variables such as cell dosage, administration route, and cell source require standardization for clinical success [188].

Finally, advanced delivery systems including ligands targeting HSCs are being developed to improve the specificity and efficacy of antifibrotic agents [189]. Affinity-based selection of peptides or antibodies may yield high-specificity delivery vehicles for small molecules, proteins, or nucleic acids [190]. In summary, overcoming therapeutic challenges in liver disease will require integrated approaches combining refined disease models, metabolic targeting, genetic engineering, cell therapy, and smart delivery technologies. Addressing disease heterogeneity, dysfunctional regeneration, and the lack of specific biomarkers will be essential to advance future treatments.

## Concluding remarks

Liver regeneration stands as a multifaceted and meticulously orchestrated biological process that engages a plethora of cellular actors and regulatory factors. Despite a surge in investigative focus over recent years, the intricate mechanisms underpinning this phenomenon remain to be fully elucidated. The translation of scientific discoveries into tangible clinical benefits is impeded by the limitations of current research models, which often fail to encapsulate the complexities encountered in actual clinical scenarios.

Most patients who undergo liver resection present with varying levels of comorbidities, which introduce a layer of variability that is not sufficiently mirrored in the experimental models. These models, while invaluable for scientific inquiry, may not accurately represent the diverse physiological states and responses of patients in clinical practice. As a result, the applicability of research findings to real-world patient care is significantly constrained. To bridge this gap, there is a pressing need to refine existing animal models to more closely reflect the clinical phenotypes observed in hepatic resection patients. Advances in this direction are essential for a deeper understanding of the regulatory mechanisms at play during liver regeneration. By enhancing the fidelity of these models, researchers can pave the way for more relevant and impactful investigations. Such endeavors aim not only to enrich the theoretical framework of liver regeneration but also to identify novel intervention points. The identification of such targets is crucial for the development of therapeutic strategies designed to mitigate the adverse effects associated with liver resection. By tailoring the models to better account for patient-specific factors, researchers can foster a more precise understanding of the regenerative process and, in turn, contribute to the evolution of personalized medicine approaches in liver surgery.

In summary, the ongoing refinement of liver regeneration models is a critical step toward unlocking the full potential of scientific research in this domain. It is through these efforts that we can hope to enhance clinical outcomes, reduce post-operative complications, and ultimately improve the quality of life for patients undergoing liver resection.
